# Functional Changes in the Glymphatic System in Children With Sensorineural Hearing Loss: A Study of Diffusion Tensor Imaging

**DOI:** 10.1155/np/4484163

**Published:** 2026-02-23

**Authors:** Jin Wang, Jiayan Zhuang, Kongqing Li, Gengbiao Zhang, Hongyi Zheng, Wenbin Zheng

**Affiliations:** ^1^ Department of Medical Imaging, Second Affiliated Hospital of Shantou University Medical College, Shantou, China, st120.cn; ^2^ Department of Radiology, Shenzhen Hospital of Integrated Traditional Chinese and Western Medicine, Shenzhen, China

**Keywords:** diffusion tensor imaging, free-water mapping, glymphatic system, magnetic resonance imaging, sensorineural hearing loss

## Abstract

**Aim:**

Diffusion tensor imaging‐analysis along the perivascular space (DTI‐ALPS) indicators and free‐water (FW) mapping derived from diffusion tensor imaging (DTI) data have been proposed as noninvasive markers of glymphatic system (GS) function. This study aimed to investigate GS function in children with sensorineural hearing loss (SNHL) with particular focus on the sensitive period of auditory development.

**Methods:**

This study enrolled 53 children with SNHL (SNHL group) and 42 age‐ and sex‐matched healthy children (healthy control [HC] group). Based on the age of 36 months, we separated the study participants into two groups: Group A (0–36 months; A‐SNHL group, *n* = 32; A‐HC group, *n* = 21) and group B (36–180 months; B‐SNHL group, *n* = 21; B‐HC group, *n* = 21). We collected their DTI image data and calculated the ALPS index for the left and right hemispheres and the fractional volume of free water in white matter (FW‐WM) and analyzed the differences between the groups. The DTI‐ALPS has several limitations, the most prominent one being the influence of microstructure. However, it has many advantages and high clinical value.

**Results:**

Compared to the HC group, the ALPS index for both hemispheres in the SNHL group was significantly lower (L: *p* < 0.001; R: *p* < 0.001), and group B exhibited the same results (L: *p* < 0.001; R: *p* < 0.001). In group A, the left‐hemisphere ALPS index of the A‐SNHL group was significantly lower than that of the A‐HC group (L: *p* = 0.002; R: *p* = 0.067). The FW imaging analysis indicated that the FW‐WM in the B‐SNHL group was significantly higher than that of the B‐HC group, and it exhibited a negative correlation with the left‐hemisphere ALPS index (*r* = −0.515, *p* = 0.017).

**Conclusion:**

Children with SNHL (especially those over 3 years old) might exhibit compromised cerebral glymphatic function, possibly attributable to clearance dysfunction and interstitial fluid (ISF) retention. Despite the recognized limitation of DTI‐ALPS, its integration with FW mapping may enhance the noninvasive indirect evaluation of glymphatic function.

## 1. Introduction

Congenital sensorineural hearing loss (SNHL) is a disorder resulting from damage to the auditory receptors, auditory nerves, or conduction pathways within the inner ear, leading to partial or complete hearing impairment [1]. Its overall prevalence is approximately 0.3% [2]. In China, the incidence of SNHL among newborns is 1‰–6‰, with an estimated 23,000 children born with congenital deafness and an additional 50,000–60,000 children experiencing delayed‐onset hearing annually [3]. Currently, substantial evidence indicates that undetected or untreated hearing loss can adversely affect a child’s language acquisition, cognitive and emotional development, and psychosocial health. Moreover, it may negatively impact fundamental life functions such as vision and motor balance, especially for children who are at their peak developmental stage [4–9].

The glymphatic system (GS) is a crucial pathway for waste metabolism in the central nervous system [10] and has been implicated in numerous neurodegenerative diseases, including Parkinson’s disease [11] and Alzheimer’s disease [12, 13]. In children, the brain GS undergoes dynamic maturation during growth and development [14, 15]. As a key network facilitating cerebrospinal fluid (CSF)–interstitial fluid (ISF) exchange and metabolic waste clearance in the brain, impaired GS function is closely associated with the pathophysiology of various pediatric neurological disorders, such as epilepsy [16–18], autism spectrum disorders [19], and pediatric Tourette syndrome [20]. These conditions are often characterized by enlargement of perivascular spaces (PVS) and a reduced analysis along the perivascular space (ALPS) index. The GS primarily participates in physiological processes, including CSF influx [21], CSF‐ISF exchange [22], and waste clearance [23] through the PVS. Dysfunction in this system can lead to the accumulation of metabolic waste, negatively affecting the nervous system. Diffusion tensor imaging‐analysis along the perivascular space (DTI‐ALPS) [24] evaluates GS functions by measuring water diffusivity within the PVS, providing a valuable indirect indicator of glymphatic clearance [25]. Wright et al. [26] reported that the DTI‐ALPS index reflects perivascular diffusivity, and it is also influenced by radial asymmetry in white matter microstructure [26]. Taoka et al. [27] further emphasized that the relationship between the ALPS index and human GS function remains unclear [27]. However, it has many advantages that other technologies cannot achieve at present and high clinical value. Free‐water (FW) mapping can accurately isolate and quantify FW in the brain, indirectly reflecting the “water retention state” of the GS [28]. Combining FW imaging and DTI‐ALPS allows for a more comprehensive evaluation of the GS’s function in children with SNHL [29].

Previous studies suggest the existence of a “sensitive period” for auditory development during the first 3 years of life [30, 31]. During this period, the brain’s neural pathways exhibit high plasticity, with rapid structural and functional development. Inputs of stimuli such as auditory and visual stimuli can have a significant impact on the brain during this period, and the absence of certain specific stimuli during the “sensitive period” may lead to irreversible corresponding brain function deficit [32–34]. Some studies have demonstrated that cochlear implantation (CI) in children with SNHL who are eligible for surgery during the peak period of brain development, especially the “sensitive period for auditory development,” can lead to better clinical outcomes and reduce the risk of severe complications, including intellectual and language impairments [34, 35]. Comprehensive investigations on the neurodevelopment in children with SNHL before and after the auditory development sensitive period are crucial for understanding their pathophysiological mechanisms. A few studies have demonstrated that children with SNHL have impaired GS function [35, 36]; however there has been a lack of research reports on the GS function in children with SNHL during the auditory sensitive period.

Our study attempts to investigate the functionality of the brain GS in children with SNHL through the application of the DTI‐ALPS index and free water in white matter (FW‐WM), with a focus on the changes in the brain GS function among children with SNHL during the auditory development sensitive period. Our study may provide novel insights into the neuropathological mechanisms of SNHL in children.

## 2. Materials and Methods

### 2.1. General Information

#### 2.1.1. Participants

This study initially enrolled 55 children with bilateral SNHL and 45 age‐ and sex‐matched healthy children with normal hearing (healthy control [HC] group) who were treated at the Second Affiliated Hospital of Shantou University Medical College between October 2012 and September 2025. Based on the predefined inclusion and exclusion criteria, two children with SNHL and three HC participants were excluded. The final cohort comprised 53 children with SNHL (SNHL group, mean age: 45.41 ± 40.64 months; male/female: 32/21) and 42 HC children (HC group, mean age: 53.74 ± 35.10 months; male/female: 24/18). The auditory development sensitive period (0–36 months) was used as a cutoff [32–34] to divide the SNHL group and the HC group into two groups: group A, aged 0–36 months (0–3 years), included 32 children with SNHL (A‐SNHL group) and 21 HC children (A‐HC group); group B, aged 37–180 months (3–15 years), included 21 children with SNHL (B‐SNHL group) and 21 HC children (B‐HC group).

#### 2.1.2. Inclusion Criteria and Exclusion Criteria for SNHL Group [37–39]

The inclusion criteria are as follows:

1. Patients under 18 years old [40].
2. Patients with SNHL with congenitally bilateral sensorineural deafness, confirmed by auditory brainstem response (ABR) results exceeding 70 dB, indicating severe or profound hearing loss. All patients were suitable for CI surgery after receiving a diagnosis of bilateral severe SNHL.
3. Patients with routine magnetic resonance imaging (MRI) and high‐resolution computed tomography (HRCT) examinations, which revealed no morphological abnormalities in the central auditory pathways, inner ear, and auditory nerve.
4. Patients with no history of head surgery, trauma, ototoxic medication use, or central nervous system disorders.

The exclusion criteria are as follows:

1. Patients who are unable to undergo MRI examination, including those with contraindications such as claustrophobia and the presence of metal implants.
2. Patients with a history of cerebral surgeries or other disorders of the central nervous system and organic brain lesions on MRI, including encephalitis or brain tumors.
3. Patients with poor image quality due to artifacts or excessive head motion (displacement >2 mm or rotation >2) in the MRI image.

### 2.2. Imaging Acquisition

All MRI scans were performed using a GE 3.0‐Tesla MRI system equipped with an eight‐channel head coil to perform routine and specialized sequence imaging scans. During the examination, participants were positioned supine with their heads immobilized to minimize motion. Moreover, a guardian was present throughout the examination to ensure participant safety, and scanning could be terminated immediately in case of discomfort or emergency.

A single‐shot spin‐echo echo‐planar imaging (SE‐EPI) sequence was used, with the following imaging parameters: Diffusion Tensor Imaging (DTI) with 15 directions: The echo time (TE) was 99.3 ms, the repetition time (TR) was 8000 ms, slice thickness = 4 mm, slice gap = 0 mm, FOV = 24 cm × 24 cm, effective matrix = 128 × 128, and the minimum and maximum *b*‐values were 0 s/mm^2^ and 1000s/mm^2^, respectively; a total of 480 images were acquired [41].

### 2.3. DTI‐ALPS Processing

**Figure 1:**
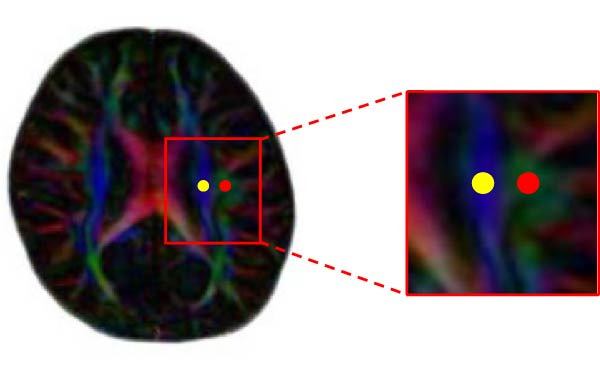
Circular ROIs with a diameter of 3 mm in the projection fibers (yellow) and association fiber (red) at the level of the lateral ventricle body.

DTI data were converted from Digital Imaging and Communications in Medicine (DICOM) format to Neuroimaging Informatics Technology Initiative (nifty) format using dcm2niigui.exe. Diffusion tensor maps, tensor maps, color‐coded fractional anisotropy (FA) maps, and automated calculations of diffusivities along the *x*‐, *y*‐, and *z*‐axes were performed utilizing DTI Studio software (version 2.30; MRI Studio Platform, Johns Hopkins University, Baltimore, MD, USA) [42]. PVS‐related diffusion was assessed by calculating diffusivity metrics adjacent to the lateral ventricles. On the FA map at the level of the body of the lateral ventricles, 3 mm‐diameter circular regions of interest (ROI) were plotted in left‐ and right‐hemisphere projection and association fibers for diffusivity measurement in three orthogonal directions (Figure 1). Two experienced neuroimaging physicians independently set ROIs, trying to keep the different data’s ROI consistent. Unaware of the grouping, they all measured the ALPS index at least three separate times, and the average of all readings was the final result.

Taoka et al. [24] reported that the ALPS index was calculated to evaluate cerebral clearing pathways utilizing the following formula, which used diffusivity ratios in four different directions.

ALPS index=MeanDxxproj,DxxassocMeanDyyproj,Dzzassoc.

Dyyproj represents the diffusivity along the *y*‐axis in projection fiber; Dzzassoc represents the diffusivity along the *z*‐axis in association fiber; Dxxproj represents the diffusivity along the *x*‐axis in projection fiber; Dxxassoc represents the diffusivity along the association fiber.

### 2.4. FW Mapping Processing

**Figure 2:**
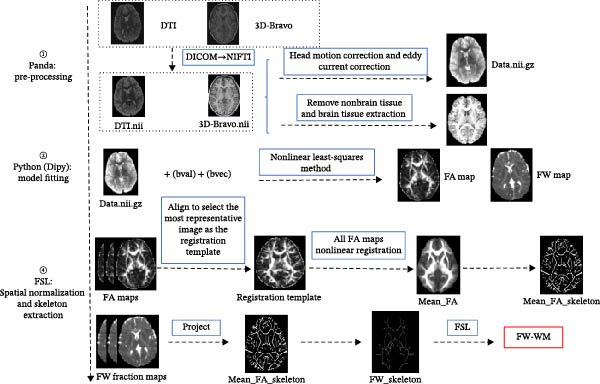
Flowchart of free‐water mapping processing.

The processing steps were as follows (Figure 2):

Preprocessing was performed using the Panda software (https://www.nitrc.org/projects/panda/). The preprocessing steps included: (1) DICOM data conversion to nifty, (2) head motion and eddy current correction, and (3) tissue segmentation based on T1 co‐registration.

Model fitting was conducted using Python with the Diffusion Imaging in Python (Dipy) (https://dipy.org/). A two‐compartment FW diffusion model was fitted to the preprocessed data [28, 43], generating a FA map and a free water volume fraction map (FW map) for each participant.

Spatial normalization and skeleton extraction were performed using Functional Magnetic Resonance Imaging of the Brain Software Library (FSL): (1) All FA maps were mutually aligned to select the most representative image as the registration template, followed by nonlinear registration to it and mean_FA_skeleton creation. (2) Individual FA and FW maps were projected onto this skeleton for group analysis.

### 2.5. Statistical Analysis

Statistical package for the social sciences software (SPSS, version 27.0) was used for statistical analysis. The Shapiro–Wilk (S‐W) test was used to ensure that the measurement data had a normal distribution and that the variance was homogeneous. Data are expressed as mean ± standard deviation (M ± SD) for readability. For data that conformed to a normal distribution, two independent samples *t*‐tests and the Pearson correlation analyses were performed. When data deviated from a normal distribution or exhibited heterogeneous variance, nonparametric Mann–Whitney *U* tests, and Spearman rank correlation analyses were employed. Sex differences between the patient group and the HC group were assessed using the chi‐square test. Age differences were evaluated using either two independent samples *t*‐tests or nonparametric Mann–Whitney *U* tests, depending on data distribution. A *p* < 0.05 was considered statistically significant.

The FW value comparison at the voxel level is based on the randomization function of FSL software and adds age as a covariate. It uses a nonparametric permutation test to perform multiple comparisons of individual parameters in the standard space of two datasets (with 5000 permutations) and simultaneously employs Threshold‐Free Cluster Enhancement (TFCE) for multiple comparisons correction. The XTRACT atlas was employed for establishing the position in anatomy of significant regions of the brain [44].

Given the exploratory nature of this study and the relatively limited sample size, and to preserve statistical power, we did not perform multiple comparison corrections on *p*‐values in any of the inter‐group comparisons conducted.

## 3. Results

### 3.1. General Information

The study included 53 children with SNHL (SNHL group) and 42 age‐ and sex‐matched healthy children with normal hearing (HC group). Using the auditory development sensitive period (0–36 months) as a boundary [32–34], SNHL and HC groups were stratified into two groups: group A, aged 0–36 months (0–3 years), included 32 children with SNHL (A‐SNHL group) and 21 HC children (A‐HC group); group B, aged 37–180 months (3–15 years), included 21 children with SNHL (B‐SNHL group) and 21 HC children (B‐HC group). No statistically significant differences were observed in participants’ age or sex between the patient groups and the HC groups. (*p* > 0.05) (Table 1).

**Table 1:** Comparison of demographics between the SNHL group and the healthy control group.

Group	*n*	Age (months) (mean ± SD)	Sex (M/F)	Age comparison (*p*‐Value)	Sex comparison (*p*‐Value)
Overall					
SNHL	53	45.41 ± 40.64	32/21	0.089^c^	0.750^a^
HC	42	53.74 ± 35.10	24/18		
Group A (0–36 months)					
A‐SNHL	32	22.03 ± 7.86	16/16	0.452^b^	0.231^a^
A‐HC	21	23.86 ± 9.59	14/7		
Group B (>36 months)					
B‐SNHL	21	75.29 ± 41.60	16/5	0.109^c^	0.057^a^
B‐HC	21	83.62 ± 23.64	10/11		

*Note:* All data are represented as mean ± SD. The threshold for significance is *p*  < 0.05.

^a^represents the use of the chi‐square test.

^b^represents the use of the two independent sample *t*‐test.

^c^represents the use of the Mann–Whitney *U* test.

### 3.2. Analysis of Left and Right Hemispheres ALPS‐Index

DTI‐ALPS analysis of the GS indicated that the ALPS index of the bilateral cerebral hemispheres in the SNHL group was significantly lower than that in the HC group. Additionally, age‐stratified analysis revealed that the ALPS index of the bilateral cerebral hemispheres in the B‐SNHL group was significantly lower than that in the B‐HC group, whereas the ALPS index of the left hemisphere in the A‐SNHL group was significantly lower than that in the A‐HC group (Table 2).

**Table 2:** Between‐group comparison of the ALPS index between each SNHL group and the HC group.

ALPS index	SNHL	HC	*p*‐Value	A‐SNHL	A‐HC	*p*‐Value	B‐SNHL	B‐HC	*p*‐Value
ALPS index‐L	1.23 ± 0.12	1.50 ± 0.20	<0.001 ^∗∗∗^	1.24 ± 0.13	1.37 ± 0.13	0.002 ^∗∗^	1.22 ± 0.09	1.63 ± 0.17	<0.001 ^∗∗∗^
ALPS index‐R	1.27 ± 0.14	1.43 ± 0.15	<0.001 ^∗∗∗^	1.30 ± 0.10	1.34 ± 0.08	0.067	1.23 ± 0.17	1.51 ± 0.15	<0.001 ^∗∗∗^

*Note:* All data are represented as mean ± SD.

Abbreviations: ALPS, analysis along the PVS; L, left hemisphere; R, right hemisphere.

^∗^ indicates *p* < 0.05.

^∗∗^ indicates *p* < 0.01.

^∗∗∗^ indicates *p* < 0.001.

### 3.3. FW Mapping Analysis

**Figure 3:**
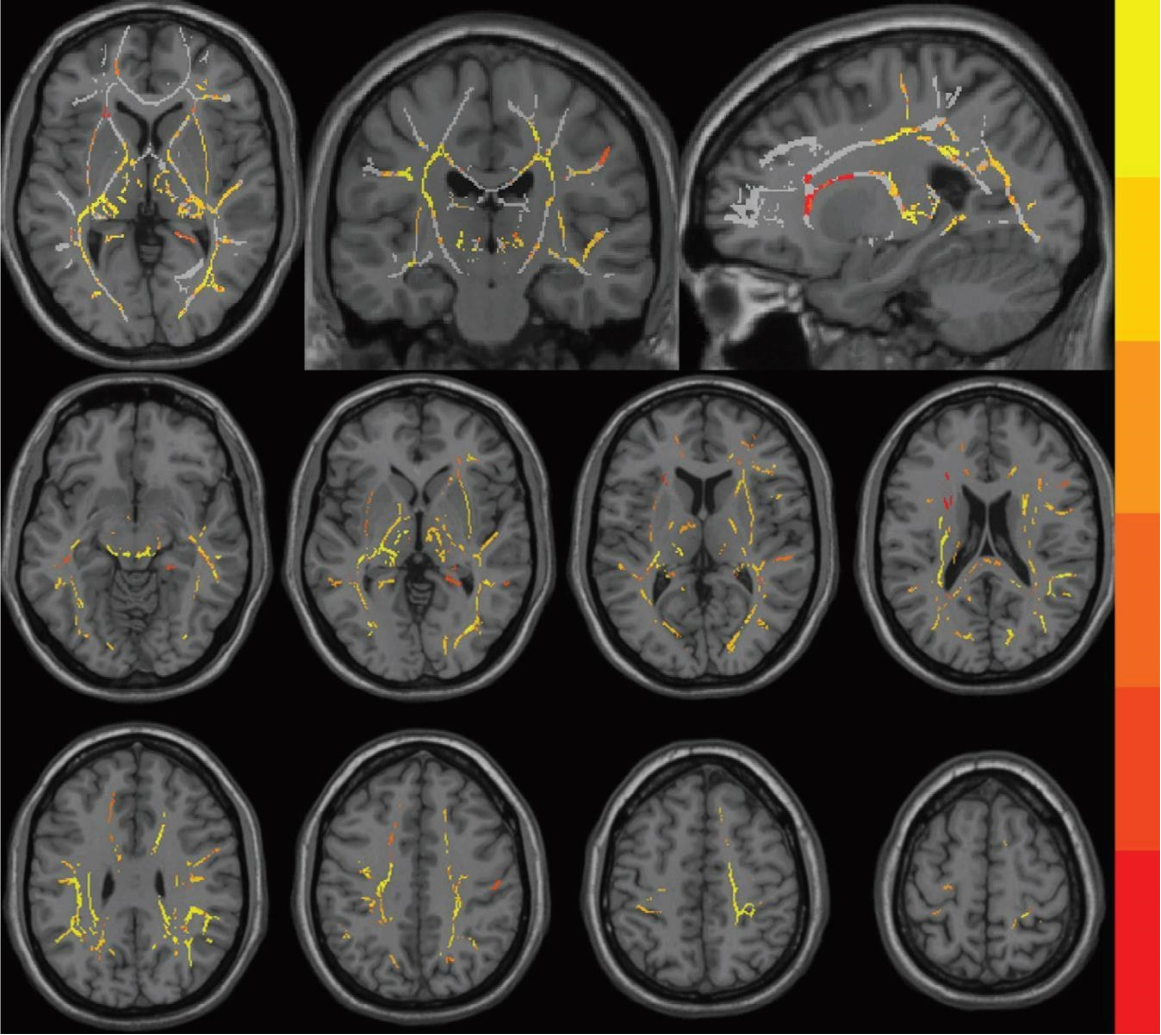
Brain region distribution of FW‐WM difference in group B. The gray areas represent the mean FA fiber skeleton, and color bars illustrate the significance of the FW‐WM difference in the respective brain regions.

At the voxel level, no significant difference was observed in the whole‐brain FW‐WM between the SNHL and HC groups. Age‐stratified analysis revealed that A‐SNHL and A‐HC groups exhibited no significant difference in their whole‐brain FW‐WM; however, the B‐SNHL group exhibited significantly higher FW‐WM in 15 regions compared to the B‐HC group (Figure 3). Refer to Table S1 for more details on the clusters. Correlation analysis revealed that only the B‐SNHL group exhibited a negative correlation between the left‐hemisphere ALPS index and the FW‐WM (*r* = −0.515, *p* = 0.017). No significant correlation was observed between the left‐ and right‐hemisphere ALPS indices and the FW‐WM in the other groups (Figure 4).

Figure 4Correlation analysis of the ALPS index and FW‐WM in each group.  ^∗^ Indicates *p* < 0.05. (a) Correlation analysis of the left‐hemisphere ALPS index and FW‐WM in the SNHL group. (b) Correlation analysis of the right‐hemisphere ALPS index and FW‐WM in the SNHL group. (c) Correlation analysis of the left‐hemisphere ALPS index and FW‐WM in the A‐SNHL group. (d) Correlation analysis of the right‐hemisphere ALPS index and FW‐WM in the A‐SNHL group. (e) Correlation analysis of the left‐hemisphere ALPS index and FW‐WM in the B‐SNHL group. (f) Correlation analysis of the right‐hemisphere ALPS index and FW‐WM in the B‐SNHL group. *r*_s_: Spearman’s rank correlation coefficient.

**Figure:**
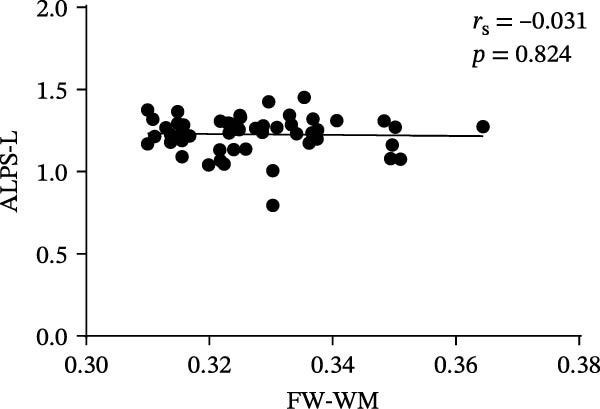
(a)

**Figure:**
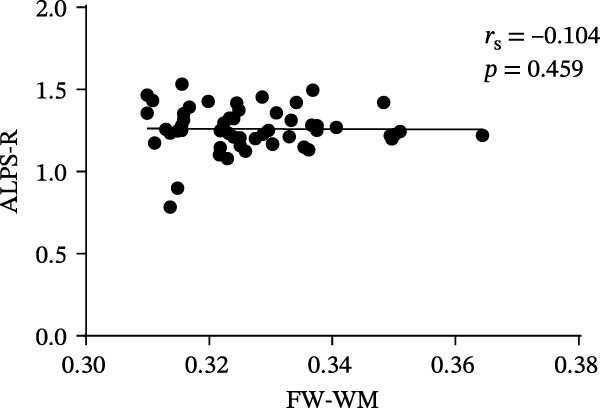
(b)

**Figure:**
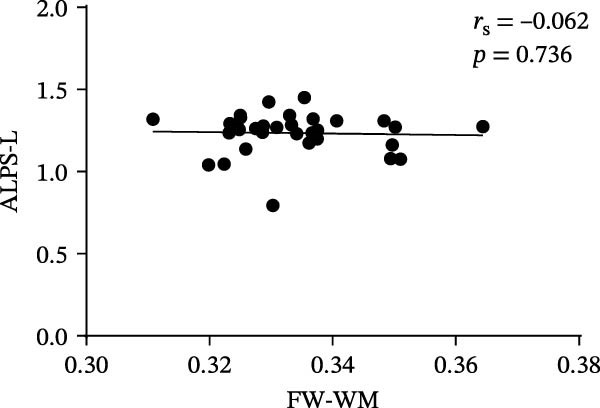
(c)

**Figure:**
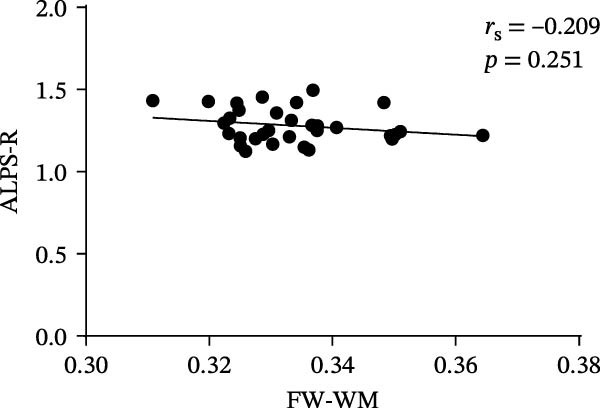
(d)

**Figure:**
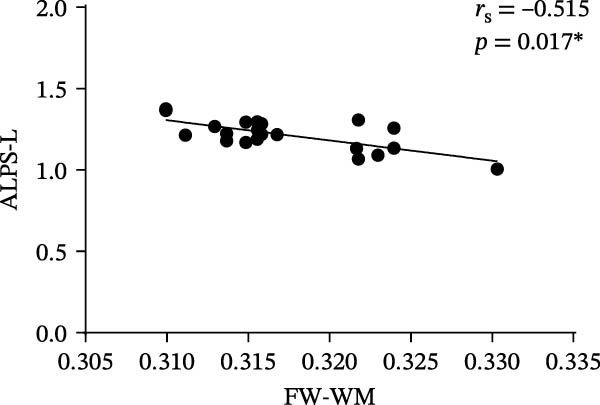
(e)

**Figure:**
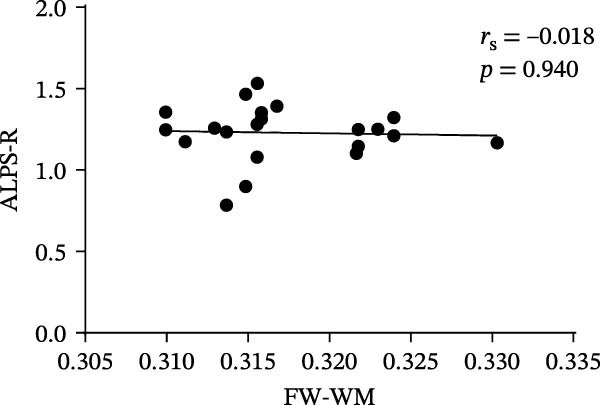
(f)

## 4. Discussion

Previous studies have indicated that auditory deprivation can lead to abnormal neuronal activity in the auditory cortex [45]. This functional alteration may result in reduced local cerebral blood flow (CBF) [23], which in turn could impair the exchange efficiency between CSF and ISF within the PVS, potentially contributing to the accumulation of metabolic waste [46]. Furthermore, prolonged hearing deprivation might be associated with microstructural WM abnormalities, including reduced myelination, axon loss, and altered fiber projections [38]. These pathological changes are particularly consequential when they occur during developmentally sensitive periods of peak neuroplasticity, potentially exerting long‐lasting effects on brain development. Herein, we evaluated the cerebral GS function in children with SNHL using DTI‐ALPS and the fractional volume of free water in cerebral white matter. To our knowledge, this is the first study to investigate GS function in children with SNHL during the sensitive period of auditory development.

Our results showed that the ALPS index in the left and right cerebral hemispheres was significantly lower in children in the SNHL group compared to those in the HC group. These findings may indirectly suggest that the GS clearance function of children with SNHL with no organic lesions in conventional MRI may have been impaired. Compared with the 0–3‐year‐ old A‐SNHL group, children older than 3 years old in the B‐SNHL group might exhibit relatively more extensive damage to the GS. We hypothesized that this phenomenon may arise from the fact that children in the sensitive period of auditory development may be more likely to compensate for and repair the damage because of the active brain remodeling function and high plasticity. However, beyond this sensitive period, auditory deprivation results in cross‐modal neural plasticity. This process facilitates the subsequent remodeling of auditory‐specific neural networks and the establishment of aberrant synaptic connections, leading to their entrenchment. The fixation of these abnormal networks poses a significant obstacle to the normal operation of the GS [47–49].

Additionally, we observed a significant increase in whole‐brain FW‐WM in children with SNHL older than 3 years old compared with age‐matched healthy children. Many previous studies have confirmed that children with SNHL will have impaired WM integrity, changes or reconstruction of brain WM microstructure, and structural networks [37, 38]. FW‐WM alterations may be associated with WM demyelination, axonal degeneration, modifications in the blood‐brain barrier, extracellular space volume, and compromised glymphatic circulation function, or a combination of these factors [50]. Based on the findings of our research, we infer that long‐term auditory deprivation in patients with SNHL over 3 years old may initiate a cascade of events: functional reorganization of the cerebral cortex, demyelination of specific neural pathways, axonal degeneration, and ISF retention due to compromised glymphatic drainage. This is consistent with the results of previous studies. Correlation analysis revealed that the ALPS index of the left hemisphere in the B‐SNHL group exhibited a negative correlation with the FW‐WM. Existing studies commonly recognized that the ALPS index may indirectly indicate the clearance function of the brain’s GS to a certain extent [51], whereas FW‐WM can represent the extracellular fluid (including brain ISF) within WM tissue [50]. Both of them offer limited but valuable insights into brain microenvironmental changes. Therefore, we can hypothesize that the impaired GS clearance function leading to the obstruction of ISF drainage may be one of the reasons for the increase of FW in the GS [41]. Conversely, no significant difference was observed in cerebral FW‐WM between children with SNHL under 3 years old and that of the HC group, which may be due to the active brain compensation repair during this period, and the degree of impaired GS clearance function could not cause a significant increase in FW volume.

Currently, research regarding the age at which CIs should be implanted in children with SNHL who are eligible for CI for a better prognosis is ongoing; however, the critical period for the development of human auditory pathways remains inadequately defined [52–54]. This study revealed that there was a more extensive impairment of the function of the cerebral GS in children with SNHL who were older than 3 years old by studying the abnormal function of the GS in children with SNHL and stratifying the age. Unfortunately, the study failed to collect relevant prognostic data. However, based on our findings, they might serve as preliminary imaging evidence suggestive of the critical period of auditory development and could, to some extent, offer a tentative reference for investigating the optimal intervention timing for children with SNHL in future clinical practice.

## 5. Conclusion

Children with SNHL might exhibit compromised GS function, with more significant damage observed in those over 3 years of age who have passed the critical period of auditory development. The underlying pathophysiology may entail impaired clearance and subsequent accumulation of ISF in the brain. The findings of our research suggest that the auditory development critical period could represent a therapeutic window for intervention. Despite the recognized limitations of DTI‐ALPS, its combination with FW mapping may enhance the noninvasive indirect assessment of glymphatic function.

## 6. Limitations

The interpretation of the DTI‐ALPS index as a specific marker of glymphatic clearance necessitates caution. First, its measurement is limited to a specific WM region adjacent to the lateral ventricle, lacking whole‐brain representativeness [55, 56]. Furthermore, it is unable to distinguish water movement inside versus outside the PVS [27]. Additionally, the direct correlation between this index and human GS function lacks thorough validation through pathophysiological studies [27, 56], thereby limiting its biological specificity. Second, the index is significantly affected by radial asymmetry in WM microstructure [26]. This means that the observed differences may reflect the combined effects of microstructural alterations and glymphatic function changes. Although our study implemented a controlled design with strict age and gender matching and included age as a covariate to mitigate such confounding, the inherent limitation of the ALPS index in disentangling microstructural from glymphatic contributions remains. Therefore, the group differences we observed should be interpreted as potentially indicative of a synthesis of both processes.

The weak correlation between whole‐brain FW‐WM and ALPS indices, as evidenced by large *p*‐values, is likely attributable to the small sample size and regional specificity, whereas the FW‐WM we measured reflects the status of whole‐brain WM, whereas ALPS indices are restricted to the periventricular WM of the bilateral lateral ventricles. These results guide future research to expand samples and focus on auditory‐specific regions. The sample size of our study is relatively small, and most of our results are exploratory, warranting dialectical discussion.

Our implementation of FW mapping aimed to improve the GS functional assessment; however, future studies need more indicators to enhance credibility [55, 57].

## Funding

This research was funded by the Basic and Applied Basic Research Foundation of Guangdong Province (Grant 2021A1515220112), the Special Fund Project for Science and Technology Innovation Strategy of Guangdong Province (Grant STKJ2023042), and the Natural Science Foundation of Guangdong Province (Grant 2024A1515011698).

## Disclosure

The creation of the manuscript and the commissioning, conception, planning, design, conduct, analysis of the work, and the preparation or editing of the manuscript were all done by the authors alone. Funders did not participate in any of the preceding steps and were solely responsible for financing publication expenses.

## Ethics Statement

The study was conducted in accordance with the Declaration of Helsinki and approved by the Ethics Review Board of the Second Affiliated Hospital of Shantou University Medical College (Protocol Code: 2022‐11, March 11, 2022). Informed consent was obtained from all subjects involved in the study.

## Conflicts of Interest

The authors declare no conflicts of interest.

## Supporting Information

Additional supporting information can be found online in the Supporting Information section.

## Supporting information


**Supporting Information** Information on brain regions with increased FW‐WM in the B‐SNHL group.

## Data Availability

Data are available upon request due to privacy/ethical restrictions.
