# Supplementary material for: Functional Changes in the Glymphatic System in Children With Sensorineural Hearing Loss: A Study of Diffusion Tensor Imaging
Source: Neural Plast. 2026 Feb 23;2026:4484163. doi: 10.1155/np/4484163 (PMC12927958; doi:10.1155/np/4484163)
Supplement: Supplementary file 1 — Supporting Information Information on brain regions with increased FW‐WM in the B‐SNHL group. [file NP-2026-4484163-s001.pdf]

Table S1. Information on brain regions with increased FW-WM in the B-SNHL group

**Cluster**	**Cluster Voxels**	**Regions**	**Peak MNI**			**P value**	**Voxels**
			X	Y	Z		
**1**	14159	Superior Longitudinal Fasciculus 3 R	23	-37	42	0.9972	1132
		Corticospinal Tract R					945
		Inferior Fronto-Occipital Fasciculus R					757
		Superior Thalamic Radiation R					753
		Arcuate Fasciculus R					741
		Forceps Major					739
		Middle Longitudinal Fasciculus R					722
		Optic Radiation R					671
		Superior Longitudinal Fasciculus 1 R					646
		Superior Longitudinal Fasciculus 2 R					501
		Frontal Aslant Tract R					492
		Anterior Thalamic Radiation R					484
		Inferior Longitudinal Fasciculus R					480
		Vertical Occipital Fasciculus R					379
		Acoustic Radiation R					356
		Forceps Minor					121
		Fornix R					63
		Cingulum subsection: Dorsal R					22
		Anterior Commissure					17
		-					4138
**2**	12691	Superior Longitudinal Fasciculus 2 L	-28	-26	-1	0.9974	1108
		Corticospinal Tract L					920
		Superior Thalamic Radiation L					793
		Inferior Fronto-Occipital Fasciculus L					693
		Arcuate Fasciculus L					625

		Middle Longitudinal Fasciculus L					581
		Anterior Thalamic Radiation L					520
		Forceps Major					440
		Superior Longitudinal Fasciculus 3 L					431
		Optic Radiation L					386
		Cingulum subsection: Dorsal L					324
		Acoustic Radiation L					298
		Vertical Occipital Fasciculus L					253
		Superior Longitudinal Fasciculus 1 L					234
		Superior Thalamic Radiation R					190
		Frontal Aslant Tract L					184
		Inferior Longitudinal Fasciculus L					163
		Anterior Thalamic Radiation R					143
		Acoustic Radiation R					67
		Anterior Commissure					25
		Uncinate Fasciculus L					18
		Forceps Minor					11
		Corticospinal Tract R					10
		Fornix L					3
		Fornix R					1
		-					4267
**3**	403	Forceps Major	-13	-39	21	0.9852	106
		-					297
**4**	345	Cingulum subsection: Temporal R	17	-43	2	0.9680	208
		Forceps Major					52
		Fornix R					11
		-					74
**5**	338	Forceps Major	-30	-34	8	0.9954	94
		Cingulum subsection: Temporal L					68
		Fornix L					39
		Acoustic Radiation L					32
		Optic Radiation L					17
		-					88

**6**	326	Superior Longitudinal Fasciculus 3 R	39	-16	30	0.9876	52
		Superior Longitudinal Fasciculus 2 R					36
		Arcuate Fasciculus R					24
		-					214
**7**	257	Anterior Thalamic Radiation L	-24	22	8	0.9630	81
		Inferior Fronto-Occipital Fasciculus L					33
		Uncinate Fasciculus L					26
		Superior Thalamic Radiation L					20
		Frontal Aslant Tract L					18
		-					79
**8**	103	Superior Longitudinal Fasciculus 3 RRadiation L	31	39	1	0.9854	27
		Inferior Fronto-Occipital Fasciculus R					18
		Forceps Minor, 11					12
		Superior Longitudinal Fasciculus 2 R					11
		-					35
**9**	99	Corticospinal Tract L	-22	-21	63	0.9770	46
		-					53
**10**	77	Superior Longitudinal Fasciculus 2 R	31	-22	36	0.9914	52
		Arcuate Fasciculus R					18
		Corticospinal Tract R					7
**11**	55	Superior Longitudinal Fasciculus 3 R	53	5	32	0.9772	34
		Arcuate Fasciculus R					2
		-					19
**12**	47	-	10	-28	24	0.9866	47
**13**	38	Superior Longitudinal Fasciculus 2 L	-33	-29	48	0.9890	5
		Corticospinal Tract L					4
		-					29
**14**	34	Cingulum subsection: Dorsal L	-17	-39	37	0.9896	3

		-					31
**15**	28	Superior Longitudinal Fasciculus 3 L	-45	-1	22	0.9616	27
		-					1

Notes: “-” indicates that the location of the corresponding regions is outside the XTRACT atlas.
